# Financing Incentives, Moral Hazard, and Welfare Loss in Iraq’s Primary Healthcare System

**DOI:** 10.34172/ijhpm.9912

**Published:** 2026-07-12

**Authors:** Dunya Al-Hayali

**Affiliations:** Sanad Organization for Economic Development, Baghdad, Iraq.

 This viewpoint assesses whether Iraq’s line-item budgeting and salaried provider model contribute to supply-side moral hazard in primary healthcare, potentially resulting in welfare loss rather than the efficiency and health gains typically associated with primary healthcare. In a resource-constrained, oil-financed system, these arrangements may undermine accountability and distort provider incentives.

 Primary healthcare centers (PHCCs) in Iraq operate under fixed, oil-financed budgets that are minimally responsive to demographic and epidemiological changes. This structure can weaken output accountability and contribute to inefficiency. While primary healthcare is generally considered a cost-effective entry point that enhances welfare through prevention and appropriate referral, Iraq’s current financing and payment arrangements may instead promote low-value utilization of scarce resources. This viewpoint develops a theory-informed argument, supported by selective evidence from the Iraqi health system, to examine this risk.

 In the 1970s and 1980s, Iraq had one of the region’s stronger health systems. Since then, war, instability, and regime change have weakened its capacity to meet rising demand and changing disease patterns.^1^ Population aging, nutritional transition, tobacco use, and disrupted immunization have increased the burden of non-communicable diseases (NCDs),^2^ raising pressure on the health budget and the need for more efficient resource allocation^3^; NCDs now account for 55% of all deaths in Iraq.^2^

 This article employs theory-informed policy analysis and a selective synthesis of health economics and Iraqi health-system literature to examine how financing arrangements influence provider incentives and welfare outcomes in primary healthcare. It further argues that free preventive care does not automatically enhance welfare when governance is weak, curative overuse is prevalent, and provider incentives are misaligned.

## Conceptual Framework: Moral Hazard in Oil-Financed Healthcare

 The conceptual framework for this analysis incorporates several related but distinct concepts from health economics and public-sector governance as they apply to Iraq’s health financing model:

1. Demand-side moral hazard. This refers to increased healthcare use when out-of-pocket costs are reduced by insurance or, in Iraq’s case, by free public provision. When patients face zero marginal cost, they may consume more services than they would if they bore the full cost, including services whose marginal benefit is lower than their marginal cost.^4^ In Iraq, this may be reflected in repeated visits and consultation-seeking for relatively minor conditions.^5-7^
2. Supply-side moral hazard. This refers to provider behavior shaped by distorted financial incentives, including overprovision, provider-induced demand, or underprovision, depending on the payment mechanism. In fee-for-service systems, providers may oversupply care; under capitation, they may undersupply; in fixed-salary systems with weak accountability, the outcome depends heavily on context.^8^ In Iraq, fixed salaries combined with weak monitoring may contribute to overprescribing, unnecessary testing, low effort, or a mixture of these behaviors.^6,7^ Although direct PHCC prescribing-audit evidence is limited, Iraq’s broader outpatient and hospital literature shows frequent empirical antibiotic use and common non-prescription dispensing, which may indicate similar risks in primary care settings.^9^
3. Allocative inefficiency. This occurs when resources are not directed to their highest value uses. In healthcare, it refers to spending on lower-value services while higher-value services remain underprovided. In Iraq, this may occur when low-value curative services crowd out higher-value preventive and primary care.^5^
4. Governance failures. These are weaknesses in oversight, monitoring, and accountability that allow inefficient behavior to persist.^10^ In Iraq, this may include limited utilization monitoring and weak performance.^7,11^
5. Soft budget constraints. These arise when organizations face limited financial consequences for inefficiency or overspending, often because they expect automatic budget support or bailouts.^12^ In Iraq, PHCCs may operate under prospective budgets that are only weakly linked to performance or outcomes.^5,6,11^

In Iraq, moral hazard primarily results from zero marginal cost to users financed by oil revenues, rather than from insurance premiums. Nevertheless, the underlying economic mechanism is similar: consumers facing zero marginal cost tend to demand more services, while providers with distorted incentives may supply inappropriate quantities or types of services. The principal distinction is that, in insurance-based systems, moral hazard imposes costs on the insurance pool and premium payers, whereas in Iraq’s oil-financed system, it imposes costs on the public budget and generates opportunity costs for other public services.

 Nyman argues that certain forms of moral hazard can be welfare-enhancing, specifically when insurance enables access to otherwise unaffordable but high-value care. This “access value” of insurance can outweigh the welfare loss from overconsumption of low-value care. However, Iraq’s case differs fundamentally: the moral hazard here does not primarily enable access to needed care (which is already free), but rather generates wasteful overconsumption and misallocation of resources. The welfare loss arises from diverting resources from high-value preventive and primary care to low-value curative services, unnecessary medications, and redundant investigations.^13^

 Within this context, welfare loss denotes the reduction in social value that occurs when healthcare resources are allocated to low-value services instead of prevention and other higher-value care. This encompasses allocative inefficiency, technical inefficiency, and fiscal waste, each with downstream consequences for health outcomes and broader public welfare. Not all observed inefficiencies in Iraq’s primary healthcare system should be classified as moral hazard in the strict economic sense; some also reflect governance failures, congestion, weak regulation, and broader institutional constraints. The central argument is that current financing arrangements may interact with these weaknesses to create conditions conducive to moral hazard and related inefficiencies.

## Iraq’s Healthcare Financing and Provider Payment Arrangements

 Iraq’s healthcare system operates under a line-item budgeting approach within the federal budget framework. The government allocates line items for salaries, operational costs, and specific expenditures, while medications and required supplies are purchased annually based on an estimate, funded by the available oil-based budget for that year.^14^ In the Ministry of Health budget, recurrent spending accounted for 80.4%, largely for wages, while 19.6% supported infrastructure, leaving limited funding for operations and medicines.^7,15^ This heavy fixed-cost burden reduces flexibility and responsiveness to changing health needs.

 In Iraq, line-item budgeting provides predictable spending, transparency, administrative simplicity, and centralized control, attributes that may be valuable in post-conflict state-building. However, this approach also incurs costs. It can exacerbate the principal-agent problem between the Ministry of Health and PHCCs, as the ministry possesses limited information on local performance while PHCCs operate under fixed budgets and salaries with minimal monitoring. This dynamic weakens incentives for efficiency and quality, allowing inefficient practices to persist.^16^ More broadly, line-item budgeting fosters rigidity, weakens performance linkages, and promotes input-focused management.

 Nevertheless, line-item budgeting is not inherently dysfunctional. In fragile and post-conflict settings, it can promote fiscal discipline, safeguard salaries, simplify administration, and maintain centralized stewardship where local capacity is limited. The central issue is whether Iraq’s current design is sufficiently flexible and accountable to support efficient primary healthcare delivery under prevailing demographic and epidemiological pressures. The assertion that primary care can absorb preventive costs while generating downstream savings at higher levels of care is more credible when budgets permit some provider- or network-level flexibility.^6^ Under a rigid line-item approach, these benefits may be difficult to achieve because local managers have limited authority to reallocate resources in response to population needs.

## Manifestations of Moral Hazard in Iraq’s Primary Healthcare Delivery

 According to the conventional theory, moral hazard in health insurance can result in welfare losses. Iraq’s system differs in that care is free at the point of use and financed by oil revenues, with no patient contribution to moderate demand. Nonetheless, the concept remains pertinent because patients may perceive services as having negligible marginal cost. Demand-side moral hazard among patients, combined with supply-side moral hazard among providers, may therefore generate dual inefficiency.^4,13^ Iraq’s PHCCs frequently face both high demand and staffing shortages^17^; rural and remote centers experience severe workforce deficits, while urban centers face overcrowding driven by unregistered walk-in demand.^1,7^ PHCCs typically operate without prior appointments or patient registration, and visits are not routinely monitored, contributing to high patient flow and weak oversight. The absence of registration or unique identifiers further limits the ability to track utilization and address inappropriate use.^18^ Consequently, spending may be diverted from higher-value uses, and primary healthcare may become more treatment-oriented than prevention-oriented. Although the financing source differs from insurance-based systems, zero marginal cost to users can still create similar incentives for overconsumption.^4^ While it could be argued that free preventive care generates welfare gains by benefiting both individuals and society, as Nyman suggests, this preventive-care welfare paradox may not apply in Iraq’s context. Overcrowding, time pressure, and excessive use of tests and medications may shift the balance toward welfare loss rather than gain.^19^ Physicians may have insufficient time for meaningful preventive counseling or chronic disease management, while PHCC activity may be dominated by acute curative consultations and prescribing.^1,20^ Thus, the theoretical welfare gains of free preventive care may not be fully realized in practice, while welfare losses from low-value curative overuse may be substantial.

 The supply-side moral hazard mechanism in Iraq’s salaried system requires careful explanation because it differs from standard economic theory. Conventional theory predicts that fixed salaries without performance incentives may lead to undersupply or low effort, as providers have limited financial incentive to increase effort.^8^ In Iraq, however, several contextual mechanisms may also encourage oversupply and overprescribing:

1. Patient pressure and demand. In some settings, patients may expect prescriptions or diagnostic tests as tangible evidence of care. Physicians may comply to avoid conflict, complaints, or prolonged consultations. In overcrowded environments, prescribing can become a lower-friction response.^21^
2. Defensive medicine. Providers may order tests or prescribe medications partly to reduce the perceived risk of complaints, litigation, or accusations of negligence. In the absence of clear clinical guidelines and strong legal protections, defensive practice may become more common.^7^
3. Weak supervision and accountability. The absence of utilization monitoring, prescription audits, or performance reviews means there are few consequences for overprescribing. Providers face no penalties for wasteful practice and no rewards for efficient practice.^10^
4. Workload dynamics. Prescribing tests and medications may be quicker than time-intensive counseling, patient education, or preventive care. Under substantial time pressure, physicians may rely more heavily on prescriptions and investigations as a practical way to manage patient flow.^1^
5. Pharmaceutical industry influence. Informal relationships with pharmaceutical representatives may encourage the prescribing of particular medications, sometimes in ways that do not fully align with clinical appropriateness.^22^
6. Cultural expectations. Societal norms equate “good care” with prescriptions and investigations. Patients may perceive physicians who do not prescribe as unhelpful or incompetent, creating social pressure to overprescribe.^21^
7. Lack of intrinsic motivation. While some providers are motivated by professional ethics and altruism, economic necessity and difficult working conditions can erode intrinsic motivation, leaving providers with neither extrinsic nor intrinsic incentives for high-quality, efficient care.^23^

Available evidence from Iraq is broadly consistent with these mechanisms. Studies report high rates of antibiotic prescribing, often for viral infections where antibiotics are inappropriate.^9^ Polypharmacy also appears common, with some patients receiving multiple medications per visit regardless of clinical indication.^24^ Diagnostic testing may follow a similar pattern, although direct Iraqi evidence on test overuse is limited.Taken together, these patterns are consistent with the possibility of supply-side moral hazard operating through the mechanisms described above.

## Provider Payment and Incentive Structures

 Iraq’s payment system is based on line-item budgeting derived from annual oil revenues. Workforce salaries are not disbursed as global budgets through individual providers such as hospitals or PHCCs; rather, they are predetermined within the Ministry of Health budget and constitute a large share of annual sectoral spending. Salaried staff, therefore, have limited extrinsic incentives to serve in remote areas or to provide high-quality care in PHCCs. Motivation may depend heavily on professional ethics and altruism, which, in turn, can be undermined by economic necessity and difficult working conditions.^23^

 Public-sector physician salaries in Iraq are low relative to private practice and regional standards, encouraging dual practice.^1^ Physicians generally earn low wages in the public sector compared to the private sector in urban areas, driving dual employment practices which can weaken commitment to PHCC work and shift effort toward private practice.^7^ At the same time, weak or inconsistent geographic incentives make remote posts unattractive despite higher costs and isolation, contributing to transfers toward cities, persistent rural understaffing, and overcrowded urban PHCCs. Formal performance incentives are also limited: salaries vary little by volume, quality, outcomes, or efficiency, and promotion may depend more on seniority than achievement. This creates a flat incentive structure that weakly rewards excellence and weakly discourages poor performance.^1^

## Regional Heterogeneity and Contextual Variation

 Iraq’s PHC system varies sharply by context. Urban PHCCs face overcrowding, overprescribing, and unnecessary testing, whereas rural centers more often face staffing shortages, limited supplies, undersupply, and quality deficits.^7,25^ In conflict-affected provinces such as Anbar, Ninewa, Salah al-Din, and Diyala, damaged infrastructure, displacement, insecurity, and weak governance mean that access and safety often take priority over concerns about moral hazard.^26^ The Kurdistan Region operates under a semi-autonomous system with somewhat different governance, some performance incentives, and stronger health information systems than those in federal Iraq, which may reduce moral hazard and welfare losses, although comparative evidence remains limited.^3^ In remote southern and western areas, severe workforce shortages mean the main challenge is undersupply and staff retention rather than oversupply control.^7^ Thus, although line-item budgeting is national, financing-driven moral hazard is most evident in urban and peri-urban PHCCs in relatively stable governorates, while remote and conflict-affected areas face more basic problems of access, staffing, and functionality. Reforms, therefore, need to be context-specific rather than uniform.

## Reform Options and a Feasible Path for Iraq’s Primary Healthcare Centers

 A shift to capitation in PHCCs could, in principle, reduce some forms of moral hazard by sharing financial risk between providers and payers, encouraging cost control, and aligning care more closely with population needs.^27^ However, capitation should not replace Iraq’s current system in the near term because its successful implementation depends on patient registration, basic risk adjustment, reliable utilization data, digital infrastructure, and stronger governance than currently exists in much of the system. Capitation also carries important risks, especially in fragile and resource-constrained settings, including underprovision of services,^28^ cream-skimming, or avoidance of high-need patients,^29^ deterioration in care quality through shorter consultations or reduced diagnostic effort,^28^ and gaming of enrolment, coding, or risk-adjustment mechanisms.^29^ Evidence from Hungary further suggests that capitation may reduce some preventive services, including screening, which is especially concerning in Iraq given its high burden of NCD and already weak preventive infrastructure.^28^

 A more feasible reform path for Iraq is therefore a phased and blended approach rather than abrupt replacement. This should combine greater managerial flexibility through global budgets for PHCC networks,^27^ selective performance-based financing for priority service and quality indicators, and blended payment models that retain a salary base while gradually introducing modest capitation and limited performance incentives.^27^ Over the longer term, broader financing reform, including social health insurance, may help strengthen sustainability and accountability.^30^ For such reforms to work, Iraq would need patient registration, quality safeguards, digital infrastructure, clear governance, referral gatekeeping, stronger pharmaceutical policy, workforce development, improved health information, and community engagement.

 As the Table shows, the most credible path is not a single ideal payment method but a sequenced combination of instruments adapted to institutional capacity and local context, drawing on the strategic purchasing and provider-payment literature.^27^

**Table:** Provider-Payment Approaches

**Financing/Payment Approach**	**Main Incentive Logic**	**Potential Advantages**	**Main Risks in Iraq’s Context**
Line-item budgeting	Controls inputs and expenditure categories	Predictability, fiscal discipline, administrative simplicity, centralized stewardship	Rigidity, weak output accountability, limited flexibility to respond to local needs
Global budgeting	Sets an overall spending envelope with some local allocation flexibility	Encourages budget discipline while allowing managerial adaptation	May still under-incentivize quality if not paired with monitoring
Capitation	Pays per enrolled person, encouraging cost control	May strengthen continuity and efficiency for defined populations	Underprovision, cream-skimming, quality deterioration, gaming where monitoring is weak
Performance-linked financing	Rewards selected outputs, processes, or quality indicators	Can improve accountability and focus attention on priority services	Gaming, data manipulation, and dependence on reliable information systems
Blended payment	Combines salary, capitation, and performance incentives	Balances income security, efficiency, and accountability	More complex to administer and monitor

## Conclusion

 This analysis indicates that Iraq’s primary healthcare system remains vulnerable if line-item budgeting and salaried payment persist without modification. Zero marginal cost to patients, weak provider incentives, and limited accountability may result in welfare loss, suggesting that reform must address not only funding levels but also incentives and accountability at the point of care. Moral hazard in primary healthcare is therefore not solely a financing issue, but also a broader challenge for a publicly funded, resource-constrained, oil-financed system.

 Priority reforms should be sequenced according to feasibility. Immediate actions include prescription monitoring, basic utilization audits, referral gatekeeping, and enhanced geographic incentives for remote service. Medium-term reforms encompass patient registration, digital infrastructure development, and piloting hybrid payment models. Longer-term reforms may involve broader provider-payment redesign and, contingent on improved institutional capacity, more extensive financing reform. As no single payment method can simultaneously balance cost control, access, quality, equity, and capacity, a phased blended approach is likely to be most practical. However, payment reform alone will not suffice; stronger governance, accountability, transparency, and responsiveness are also essential. Without such reforms, Iraq’s primary healthcare system may continue to generate welfare losses rather than the efficiency and health gains that primary care is intended to deliver.

## Disclosure of artificial intelligence (AI) use

 Grammarly was used solely for language editing.

## Ethical issues

 Not applicable.

## Conflicts of interest

 Author declares that she has no conflicts of interest.
